# Relationship Between Psychological Responses and the Appraisal of Risk Communication During the Early Phase of the COVID-19 Pandemic: A Two-Wave Study of Community Residents in China

**DOI:** 10.3389/fpubh.2020.550220

**Published:** 2020-09-30

**Authors:** Zheng Jin, Kai-bin Zhao, Yan-yu Xia, Rui-jun Chen, Huan Yu, Timothy Tamunang Tamutana, Zheng Yuan, Yi-Ming Shi, Hanna Yeshinegus Adamseged, Marina Kogay, Gyun Yeol Park

**Affiliations:** ^1^International Joint Laboratory of Behavior and Cognitive Science, Zhengzhou Normal University, Zhengzhou, China; ^2^College of Education, Shanghai Normal University, Shanghai, China; ^3^School of Education, Henan University, Kaifeng, China; ^4^College of Education, Gyeongsang National University, Jinju, South Korea

**Keywords:** COVID-19, emotion, anxiety, preventive measures, risk communication, risk perception, mental health, longitudinal data

## Abstract

The novel coronavirus disease (COVID-19) has affected hundreds of millions of people worldwide. Data collection in the ascending phase is crucial to address a rapidly evolving crisis by helping us understand the uncertain relationship between risk communication and psychological responses. Data were collected from 26 January 26, 2020, until February 17, 2020, with a mean test–retest interval of 16 days. A total of 846 adults from four residential communities in high-risk areas (Wuhan city) and low-risk areas (Zhengzhou city) were invited to complete a set of Internet-based questionnaires measuring the adoption of preventive behaviors, appraisal of risk communication, anxiety level, and susceptibility to emotional contagion. At the baseline assessment (Wave 1), 58 withdrew from the study, and 788 (433 females) completed the questionnaires. At the Wave 2 survey, 318 (185 females) adults from Wave 1 were retained. The results from cross-lagged models demonstrated reciprocal negative associations between anxiety and risk communication and between the appraisal of risk communication and the adoption of preventive behaviors. In addition, a higher appraisal of risk communication in the initial period of the outbreak mitigated the respondents' susceptibility to emotional contagion later on. Susceptibility to emotional contagion was positively associated with preventive behaviors taken. Furthermore, multiple-group structural equation modeling suggested that risk communication was more likely to affect the susceptibility to emotional contagion of people on the frontline of the outbreak than people living in low-risk areas. This study demonstrated the importance of risk communication aimed at encouraging appropriate countermeasures against virus outbreaks.

## Introduction

On March 11, 2020, the World Health Organization declared coronavirus disease 2019 (COVID-19) a pandemic. As of March 28, 2020, a total of 571,678 confirmed COVID-19 cases, and 26,494 deaths had been reported worldwide. Medical interest in COVID-19 has been considerable [e.g., (1)]. Mental health issues that coincide with emerging epidemics and the appropriate behaviors to adopt to avoid infection are rarely examined (2).

Viral disease infections usually come from ordinary contact with people, and outbreaks can trigger severe public panic. In particular, novel, exotic threats raise anxiety levels higher than more familiar threats do (3, 4). Studies during the severe acute respiratory syndrome (SARS) epidemic in 2003 showed that diagnosed patients, suspected patients, and normal people experienced intense fear or nervousness about the event, and their anxiety increased significantly (5). Moreover, emotions are extremely vulnerable during public health emergencies (6), and the fear of a vague and terrifying new illness might spiral into dangerous skepticism through emotional contagion, which refers to the phenomenon of having one person's emotions directly trigger similar emotions in other people [c.f., (7)]. It was predicted in 2018 that the next major outbreak might not be due to a lack of preventive technologies but to emotional contagion, which could erode trust in government, causing serious economic and social disruption (8).

Although many studies have pointed out that high risk perception may lead to excessive preventive behavior and bring more emotional problems (9), in the early stages of major public health emergencies, increasing the level of risk perception is still a necessary means to combat viral spread. A recent study estimates that improving the rates of handwashing by travelers passing through only 10 of the world's leading airports could significantly slow a global disease by 69% (10). It is noteworthy that preventive behavior is also affected by emotional state. A survey of earthquake victims indicated that preparedness behavior could be predicted by fear and anxiety (11). Leung et al., studying public health emergencies, reached a consistent conclusion, finding that anxiety level is positively correlated with preventive measures taken (12, 13). It may be that individuals with higher levels of anxiety hold higher risk perceptions (14), so they take preventive measures as a means of coping with anxiety in risk events (15). Similarly, individuals with high susceptibility to emotional contagion are also more likely to be affected by risk information (16), thereby alleviating emotional problems through preventive measures (17).

Risk communication refers to the exchange of real-time information, advice, and opinions between experts and people facing threats to their physiological, economic, or social well-being. On the one hand, effective, timely and credible risk communication is essential to containing fear and public threats (18) as well as promoting preventive behaviors, especially in the early phase of risk events, because this increases perceived risk (19). On the other hand, psychological traits may in turn give rise to bias against the local crisis management system. For example, people with higher levels of anxiety may be more likely to overreact to policies (20). Individuals who are susceptible to negative emotions may more easily hold beliefs that conflict with government advice or regulations, thus jeopardizing public health measures [e.g., (7)]. Governments have the hard job of explaining dangers and advising people how to act without raising alarm, and the uncertain relationship between risk communication and psychological response needs to be investigated.

Some recent studies have also discussed the relationship between anxiety and emotional contagion. Anxious individuals tend to catch emotions from others, and emotional susceptibility has the unfavorable effect of making the person more anxious [e.g., (21); for review, (22)]. In summary, the existing research cannot accurately explain the interaction between multiple factors and their multidimensional causality. COVID-19 is an unprecedented experience for many people. Therefore, in the early stage of the epidemic, public emotions and behaviors in response to the epidemic may change rapidly with the exponential growth rate of the outbreak while being influenced by risk communication. What is the public reaction to epidemic outbreaks in the early phase? How does the effective exchange of real-time risk information impact them over time? What are the characteristics of these effects under different risk intensities? The present study examined the temporal relationships among behavioral and emotional responses to COVID-19 and the attitudinal responses to risk communication. A 2-way design was employed. We hypothesized that the adoption of preventive behaviors, emotional anxiety, and susceptibility to catch emotions were associated with the appraisal of risk communication as the pandemic developed.

## Materials and Methods

### Sampling and Data Collection

Three research assistants and five residential community staff members participated in the survey distribution. Invitations containing links to this Internet-based survey and quick response codes were sent to local communities in Wuhan and Zhengzhou via messenger apps with the group function. Data were collected from January 26, 2020 (at which time 30 provinces launched their first-level response to this major public health emergency in China, and 20 cases had been confirmed in Zhengzhou, China, making it a low-risk area, while 63 deaths and 698 cases had been confirmed in Wuhan, China, making it a high-risk area), with 4-day duration until February 17, 2020 (154 cases had been confirmed in Zhengzhou; 1,381 deaths and 42,752 cases had been confirmed in Wuhan), with 3-day duration. The mean test–retest interval was 16 days (*SD* = .82), with a range of 14 to 18 days. The data collected by these surveys thus covered the ascending phase of the outbreak (23).

A total of 846 adults from four residential communities (i.e., two communities in Hanyang, which is an urban administrative district of Wuhan, and two communities in Erqi and Zhongyuan, which are also the main administrative districts of Zhengzhou) were invited to complete a set of questionnaires. Wuhan and Zhengzhou, as the China national central cities, are at a similar level in terms of leading, developing, and performing tasks in politics, economics, and culture (24). Sociodemographic data were collected on sex, age, education, current health status, diagnosis with COVID-19, suspicion of COVID-19, having contact with a confirmed patient, and having contact with a suspected patient.

The questionnaires used a forced response mode that required respondents to answer all the questions before proceeding, but respondents could withdraw from the study at any time. Participants completed the questionnaires after giving online informed consent. The last six digits of the participant's phone number were used as their unique ID. We used the phone numbers, IP addresses recorded by the network server, and manual verification as the means of data matching. To ensure participant confidentiality, we purposely analyzed the data only in aggregate and did not perform individual program analyses.

At the baseline assessment (Wave 1), 58 withdrew from the study, and 788 (433 females, mean_age_ = 34.66; *SD* = 7.34, 500 from Zhengzhou and 288 from Wuhan) completed the questionnaires. At the Wave 2 survey, 318 adults from Wave 1 were retained. Of these respondents, four participants gave arbitrary answers on age in both waves (e.g., 888), which were treated as missing data and handled by mean imputation. The final samples of Zhengzhou and Wuhan were different in age, *t* (316) = −5.31, *p* < 0.01; and education, χ^2^(4) = 38.99, *p* < 0.01. This is mainly manifested in the fact that the Wuhan group is older and the Zhengzhou group has more people with a master's degree or above. In addition, a suspected case was reported in Wuhan (Table 1). Those who we were unable to retrospectively follow up fell into attrition. In many longitudinal studies, observations across waves can be missing for various reasons, and the attrition rate for web-based surveys is especially high (25). Another reason for our high attrition rate may be that we use the forced response mode; people will stop working on the survey if they are asked questions they do not wish to answer (26), although some of our questions included a “no answer” option.

**Table 1:** Sociodemographic characteristics of participants at baseline.

**Baseline characteristic**	**Respondents from Zhengzhou *N = 175***	**Respondents from Wuhan *N = 143***
*Age*	yr. 30.28 ± 7.52	yr. 34.63 ± 6.94
*Education level*		
Under high school	1	3
High school	4	3
College or B.A.	109	128
M.A.	44	8
Ph.D.	17	1
*Current health status*
Excellent	58	40
Good	96	78
Average	9	10
Fair	6	9
Poor	6	6
*Confirmed case ^a^*		
No	173	135
Do not answer	2	8
*Suspected case*
Yes	0	1
No	172	139
Do not answer	3	3
*Having contact with diagnosed case*
Yes	0	0
No	156	135
Do not answer	19	8
*Having contact with suspected case ^*a*^*
No	173	137
Do not answer	2	6

a *Reflects no “Yes” respondent to this question*.

Several analyses were performed to test whether there was a systemic pattern to the participant loss. The chi-square test showed a significant linear-by-linear association (*p* < 0.001), suggesting that the attrition rate decreased with increasing education level. Attrition at Wave 2 was lower among the younger participants, *t*(624) = −7.65, *p* < 0.01 [Levene's test indicated unequal variances (*F* = 9.09, *p* = 0.003), so the degrees of freedom were adjusted from 786 to 624]. Higher age may be regarded as a predictor of withdrawal due to less frequent Internet usage [(27); c.f., (28)]. The difference between the attrition and retained proportions by sex, χ^2^(1) = 2.42, *p* > 0.05, current health status, χ^2^(4) = 1.92, *p* > 0.05, and all other studied variables did not reach statistical significance, *ps* > 0.05 for all.

### Measures

#### Adoption of Preventive Behaviors (APB)

Eight questions based on recommendations from the China Center for Disease Control and Prevention (CDC) guidelines were developed. Sample items of preventive measures included “Did you wash your hands after sneezing, coughing, or cleaning your nose in the past three days?” All eight behavior items were rated on a four-point scale, ranging from 1 “Not at all” to 4 “Always.” The total frequency of APB was calculated by summing the scores of all 8 items. APB had Cronbach's alpha values of 0.75 and 0.81 for the Wave 1 and Wave 2 data, respectively.

#### Appraisal of Risk Communication (RMC)

A six-item scale was used to assess appraisal of risk communication. It was designed to reflect opinions on information distribution and openness of information [(29), e.g., “With regard to the distribution of information by the health authorities to the public in your country, do you agree or disagree that it has generally been sufficient?” Or “Do you agree or disagree that you have had the chance to express your personal views and concerns to the authorities if you wanted to?”]. The items are scored on a six-point scale, with higher scores indicating more positive appraisal (based on these replies: “strongly disagree,” “disagree,” “not sure but probably disagree,” “not sure but probably agree,” “agree,” “strongly agree”). The questionnaire was shown to have acceptable validity and high internal consistency. Cronbach alpha values in our sample were 0.87 for both waves.

#### Anxiety Level

Anxiety were assessed using the Zung Self-Rating Anxiety Scale (SAS) (30), which consists of 20 items. Questions 1–5 represent the emotional symptoms of anxiety of which question 5 is a reverse-scored item, while questions 6–20 represent the physical symptoms of anxiety [e.g., (31)]. Responses to each item range from 1 (“a little of the time”) to 4 (“most of the time”), with higher scores indicating increased levels of anxiety. Emotional symptoms of anxiety were the main concern in this study (e.g., “I feel more nervous and anxious than usual”). Reliability coefficients were good for both Wave 1 (Cronbach α = 0.82) and Wave 2 (Cronbach α = 0.83) samples in the current study.

#### Susceptibility to Emotional Contagion (SEC)

The Emotional Contagion Scale for Public Emergency (ECS-PE) (32) is a self-report scale for assessing the susceptibility to catch emotions, especially generated in public emergency events (e.g., When public emergency happens, I panic if others around me panic). It is a revised version of the Emotional Contagion Scale (33) and consists of 15 items that a person endorses on a five-point scale (ranging from 1 “strongly disagree” to 5 “strongly agree”). Scores are generated by adding the item scores. This scale had Cronbach α values of 0.90 and 0.91 for the Wave 1 and Wave 2 data, respectively.

## Plan of Analysis

Repeated-measures analyses of variance (ANOVAs) were conducted to determine time and risk effects over the two waves of the study. We also computed descriptive statistics for all study variables and bivariate correlations among them using SPSS 20.0. Then, cross-lagged models were tested by structural equation models with the robust maximum likelihood estimation using MPlus version 7 (34). Finally, to additionally assess whether the cross-lagged associations varied by group (i.e., Zhengzhou vs. Wuhan, which represents risk level), we ran multigroup structural equation models. The following steps were conducted: (1) unconstrained multiple-group model, in which the same correlation of paths was tested without constraints across groups; and (2) constrained multiple-group model, where correlation paths were constrained to be equal across groups.

Model fit was examined by the chi-square statistic (χ^2^), the comparative fit index (*CFI*), the Tucker–Lewis Index (*TLI*), the root mean square error of approximation (*RMSEA*), and the standardized root mean square residual (*SRMR*). Good model fit is indicated by a nonsignificant χ^2^ (35), a *CFI* and/or *TLI* between.90 and 1.00 (36), an RMSEA of.10 or lower (37), and an SRMR of.10 or lower (35).

## Results

### Descriptive Statistics and Bivariate Correlations

Inspection of Mahalanobis *d*^2^ values indicated that there were six outliers in our sample. Omitting the outliers gave the same results as not. Repeated-measures ANOVA revealed a significant main effect of time on APBs, *F*_(1, 316)_ = 48.67, *p* < 0.001, η_*p*_^2^ = 0.13, and a significant main effect of risk level (i.e., Zhengzhou vs. Wuhan) on APBs, *F*_(1, 316)_ = 10.83, *p* < 0.01, η_*p*_^2^ = 0.03, and on anxiety level, *F*_(1, 316)_ = 31.94, *p* < 0.001, η_*p*_^2^ = 0.10. A significant risk × time interaction on susceptibility to emotional contagion (SEC) was found, *F*_(1, 316)_ = 7.26, *p* < 0.01, η_*p*_^2^ = 0.02. Simple effect analyses revealed that SEC decreased significantly for participants in Zhengzhou, *F*_(1, 317)_ = 4.34, *p* < 0.05, η_*p*_^2^ = 0.02, but did not change with the development of the epidemic for participants in Wuhan. Although the ANOVA showed that the means were significantly different, the effect size was small to modest. Table 2 presents bivariate correlations among Wave 1 and Wave 2 variables, which indicated considerable stability in autoregressive correlation between all studied variables, and revealed cross-lagged relations between appraisal of risk communication and anxiety. The cross-sectional intercorrelations among all variables were similar across Wave 1 and Wave 2.

**Table 2:** Descriptive statistics and bivariate correlations between measured variables (*N* = 318).

		**T1_APB**	**T1_RCM**	**T1_Anxiety**	**T1_SEC**	**T2_APB**	**T2_RCM**	**T2_Anxiety**	**T2_SEC**
1	T1_APB	1							
2	T1_RCM	.375^**^	1						
3	T1_Anxiety	−.298^**^	−.246^**^	1					
4	T1_SEC	.264^**^	0.04	−.215^**^	1				
5	T2_APB	.675^**^	.371^**^	−.267^**^	.269^**^	1			
6	T2_RCM	.354^**^	.675^**^	−.288^**^	0.075	.394^**^	1		
7	T2_Anxiety	−.286^**^	−.278^**^	.683^**^	−.223^**^	−.306^**^	−.289^**^	1	
8	T2_SEC	.180^**^	−.062^**^	−.154^**^	.814^**^	0.275^**^	−0.005	.−.167^**^	1
Zhengzhou	*M*	23.47	28.51	10.11	52.37	25.10	28.41	9.75	51.43
	*SD*	4.50	4.88	3.51	9.66	4.49	4.80	3.65	10.54
Wuhan	*M*	25.17	28.44	12.01	52.88	26.43	27.97	11.80	52.47
	*SD*	4.71	4.52	3.31	9.76	4.25	5.18	3.08	9.85

*T1, Wave 1; T2, Wave 2*.

** *Correlation is significant at the 0.01 level (two-tailed). APB, adoption of preventive behaviors; RCM, appraisal of risk communication; SEC, susceptibility to emotional contagion*.

### Cross-Lagged Model

**Figure 1:**
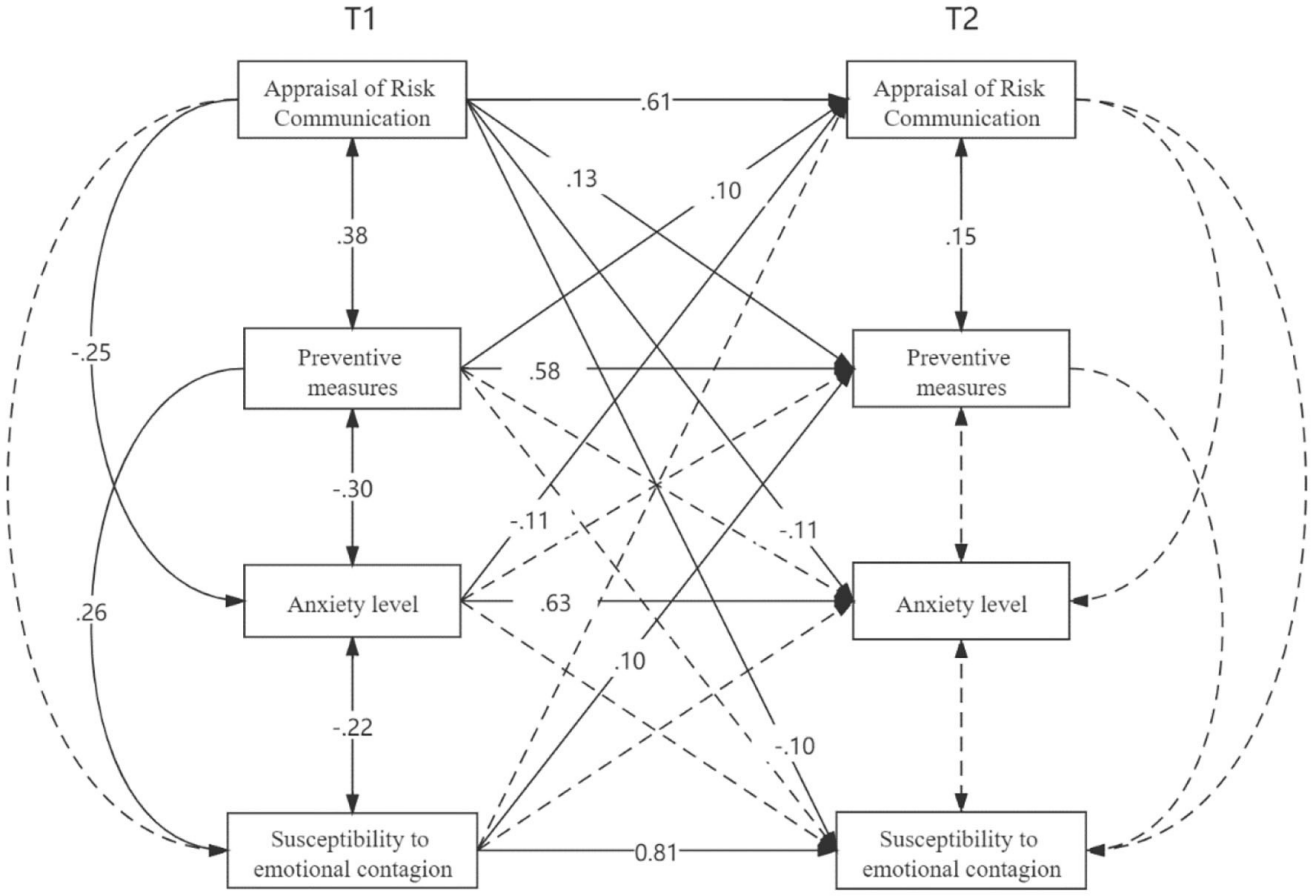
Two-wave cross-lagged model. The values reported are the standardized coefficients. The pathways that were nonsignificant remained in the model, but for the simplicity of interpretation, they are not presented in the figure.

The model with full cross-lagged paths demonstrated an acceptable fit to the data. Given the sensitivity of the χ^2^ statistic to sample size (35), it was not surprising that the test was significant (χ^2^ = 39.781, *p* < 0.001, *df* = 16). However, Wheaton et al. (38) maintain that a χ^2^/*df* ratio below five supports a favorable conclusion about fit in large sample models. In this study, this criterion is solidly met, χ^2^/*df* = 2.49, *p* < 0.01; *CFI* = 0.97, *TLI* = 0.92, *RMSEA* (90% CI) = 0.07 (0.04–0.10), *SRMR* = 0.05. The autoregressive paths between Wave 1 and Wave 2 for APB, β = 0.58, *SE* = 0.06; RCM, β = 0.61 *SE* = 0.05; anxiety, β = 0.63, *SE* = 0.04; and SEC, β = 0.81, *SE* = 0.02, were all significant, *ps* < 0.01. After controlling for demographic variables (i.e., gender, age, education level and health status), one positive pathway from RCM to later APB, β = 0.13, *SE* = 0.05, and one negative path from Wave 1 RCM to Wave 2 SEC, β = −0.10, *SE* = 0.03, were revealed. Two reciprocal associations between RCM and anxiety, β*s* = −0.11 for both directions, and RCM and ABP, β = 0.10 and 0.13, for two directions respectively, *ps* < 0.05, were also detected. The whole model accounted for 47.9, 48.1, 49.2, and 67.5% of the total variance in Wave 2 APB, RCM, anxiety, and SEC, respectively. The standardized path coefficients are presented in Figure 1. Age and gender have an effect on the susceptibility to emotional contagion (SEC). The older the age, the greater the SEC, β = −0.08, *SE* = 0.03, and women hold more susceptibility than men, β = −0.07, *SE = 0.03*.

### Multigroup Structural Equation Model

The chi-square of the baseline model (unconstrained) is 0 because it is a saturated model. A significant difference in chi-square indicates non-equivalence across groups, Δχ^2^ = 38.75, Δ*df* = 22, *p* < 0.05, suggesting that one or more paths are different across group from areas in different risk level. Further, Wald tests were used to examine differences among the cross-lagged paths between the two groups and revealed that the coefficient of the paths from Wave 1 RCM to Wave 2 SEC was significantly higher for Wuhan than those in Zhengzhou, Wald(1) = 7.14, *p* < 0.01. The association of RCM with SEC reached a significant level for Wuhan, β = −0.20, *p* < 0.001, but not for Zhengzhou, β = −0.05.

## Discussion

This study sought to gather a snapshot of the attitudinal and behavioral responses during the early stages of the COVID-19 epidemic. The results showed that the level of anxiety of people in high-risk epidemic areas is significantly higher than that in low-risk areas. A virus that is thought to be highly contagious, lockdown control, and disturbances in people's living conditions are all factors that cause mental problems in epidemic areas in the short term. It is not surprising that as the epidemic progressed, respondents adopted more preventive measures, and people in high-risk epidemic areas also took preventive measures to a greater extent, indicating a high-risk perception.

Without information, people may start speculating and “filling in blanks” on their own. This often results in increased susceptibility to emotional contagion (SEC), which is a catalyzer that accelerates the spread of rumors [e.g., (39)]. The finding that the susceptibility was significantly lower in low-risk areas suggests to some extent that the increased susceptibility caused by the emergency was alleviated by the gradually disclosed information, even though participants living in high-risk areas did not change in any way. This suggestion was further verified by cross-lagged panel analysis. The initial appraisal of risk communication was predictive of later susceptibility to emotional contagion, and such an association exhibited a greater impact on people of the frontline of the outbreak (i.e., Wuhan). Previous studies have pointed out that effective risk communication can mitigate susceptibility and is an important means to relieve public anxiety [e.g., (40)]. However, this study demonstrated a reciprocal association between anxiety and risk communication, reflecting that the emotional aspect may create resistance to risk communication.

Some previous research that has focused on responses to other respiratory infectious disease epidemics (RIDEs) has examined factors that motivate people to adopt preventive measures. For example, Lee-Baggley and colleagues found that people high in empathic responsiveness (e.g., listening to others' feelings about SARS) were more likely to take health precautions (41). Consistent with these findings from cross-sectional studies, individuals who were more susceptible to emotional contagion early on were more likely to engage in preventive behaviors later. However, not all mood states affect behavior. Compared with susceptibility, initial anxiety did not predict later adoption of preventive measures. A possible explanation is that in the early stage of an epidemic, when the threat is highly uncertain, cognitive risk responses may be optimal for driving increasingly suitable behavior as the epidemic evolves (42). Emotional contagion occurs at more conscious levels [for review, (43)]. Anxiety generally involves less intense cognitive components than susceptibility to emotional contagion and thereby is less likely to predict behavioral change.

The respondents' appraisal of risk communication predicted the extent to which they would engage in preventive behaviors, which indicates that preventive measures are undoubtedly closely related to the effective and timely transmission of epidemic-related information. The results also revealed the effect of changes in behavior on the changes in the appraisal of risk communication. If an action is believed to reduce risk, people who take the action will lower their perceived risk (19, 44), leading to decreased sensitivity to risk information. We did not find any correlation between anxiety and susceptibility to emotional contagion, although it is evident from various findings that anxious individuals tend to catch negative emotions from others [e.g., (21)]. Given the evidence presented in this study, however, it seems clear that anxiety was unrelated to susceptibility to emotional contagion as measured on a bipolar scale that measures reactions to both positive and negative emotions. In addition, we extracted emotional symptoms of anxiety from the more general Zung Self-Rating Anxiety Scale (SAS) for screening anxiety, which is different from the State Trait Anxiety Inventory (STAI) used in some previous epidemic studies [e.g., (45)]. In line with these studies, the anxiety level remained low throughout the pandemic, suggesting that a low level of anxiety has little effect on behavioral or emotional responses toward COVID-19.

A few limitations to this study are worth noting. First, with regard to the measurements we used, a set of questions measuring the extent to which a respondent adopted preventive behavior may not fully reflect all the preventive measures required to prevent infections. Second, the results may have limited generalizability because this community sample was limited in its diversity, as a majority of the sample consisted of middle-aged and healthy people. Lastly, this study used district (i.e., Wuhan and Zhengzhou) as an indicator of risk. Although some demographic variables were controlled, there are some unobserved heterogeneity (e.g., income and occupation) may limit conclusions of the study. Nevertheless, to our knowledge, these data provide some of the first follow-up data regarding mental health during the COVID-19 outbreak. Data collection in the ascending phase is crucial to deal with a rapidly evolving crisis. More harm is done by officials trying to avoid panic by withholding information or overreassuring the public than is done by the public acting irrationally in a crisis. Precrisis planning should assume that an open and honest flow of information will be established. This study demonstrates the importance of the disclosure of information aimed at encouraging appropriate countermeasures against virus outbreaks.

## Data Availability Statement

The datasets presented in this article are not publicly available due to the fact they containing sensitive information that could compromise research participant privacy/consent. However, they are available on reasonable request from the corresponding author. Requests to access the datasets should be directed to psychlab@zznu.edu.cn.

## Ethics Statement

The study protocol, including questionnaires, was reviewed by the Ethics Committee of Zhengzhou Normal University (approval no. 2020EM-01) and Institutional Review Boards of University of California at Davis (IRB ID 1561876-1).

## Author Contributions

ZJ conceived of the presented idea and planned the experiments. ZJ took the lead in writing the manuscript with input from all authors. K-bZ and Y-yX analyzed the data and contributed equally to this article. Y-MS, R-jC and ZY contributed to sample preparation. GYP provided financial support that are necessary for this study. All authors read and approved the final version of the manuscript.

## Conflict of Interest

The authors declare that the research was conducted in the absence of any commercial or financial relationships that could be construed as a potential conflict of interest.
